# Correction: Huynh et al. Spike Protein Impairs Mitochondrial Function in Human Cardiomyocytes: Mechanisms Underlying Cardiac Injury in COVID-19. *Cells* 2023, *12*, 877

**DOI:** 10.3390/cells13221865

**Published:** 2024-11-11

**Authors:** Tin Van Huynh, Lekha Rethi, Ting-Wei Lee, Satoshi Higa, Yu-Hsun Kao, Yi-Jen Chen

**Affiliations:** 1 International Ph.D. Program in Medicine, College of Medicine, Taipei Medical University, Taipei 11031, Taiwan; d142109010@tmu.edu.tw (T.V.H.); yjchen@tmu.edu.tw (Y.-J.C.); 2 School of Biomedical Engineering, College of Biomedical Engineering, Taipei Medical University, Taipei 11031, Taiwan; lekhar@tmu.edu.tw; 3 International Ph.D. Program for Biomedical Engineering, Taipei Medical University, Taipei 11031, Taiwan; 4 Division of Endocrinology and Metabolism, Department of Internal Medicine, School of Medicine, College of Medicine, Taipei Medical University, Taipei 11031, Taiwan; b8801138@tmu.edu.tw; 5 Division of Endocrinology and Metabolism, Department of Internal Medicine, Wan Fang Hospital, Taipei Medical University, Taipei 11696, Taiwan; 6 Cardiac Electrophysiology and Pacing Laboratory, Division of Cardiovascular Medicine, Makiminato Central Hospital, Okinawa 901-2131, Japan; higa@haku-ai.or.jp; 7 Graduate Institute of Clinical Medicine, College of Medicine, Taipei Medical University, Taipei 11031, Taiwan; 8 Department of Medical Education and Research, Wan Fang Hospital, Taipei Medical University, Taipei 11031, Taiwan; 9 Division of Cardiovascular Medicine, Department of Internal Medicine, Wan Fang Hospital, Taipei Medical University, Taipei 11031, Taiwan

## Text Correction

There was an error in the original publication [1]. In Section ‘2.8. Intracellular Ca^2+^ Measurements’, the text, ‘thapsigargin (100 nM)’ was wrong. A correction has been made and the wrong text was replaced with ‘thapsigargin (2.5 µM)’. The corrected Section appears below.

### 2.8. Intracellular Ca^2+^ Measurements

Fura-2 was used for measuring cytosol Ca^2+^ levels as described previously [19]. Briefly, AC16 cells treated with S1 for 24 h or 72 h were incubated with fura-2-acetoxymethyl ester in black-wall 96-well plates (5 μM; Life Technologies, Carlsbad, CA, USA) in F12-DMEM medium for 30 min at 37 °C and 5% CO_2_. Fura-2 fluorescence was read using a BMG multi-mode microplate reader (BMG lab tech, Allmendgrün 8, 77799 Ortenberg, Germany) with dual-excitation wavelengths of 340 and 380 nm. After measuring the baseline cytosol Ca^2+^ (cells were incubated in Ca^2+^-free Tyrode solution) for 2 min, thapsigargin (2.5 μM) was added to block endo/sarcoplasmic reticulum (SR) Ca^2+^-ATPase to evaluate Ca^2+^ release. As soon as the Ca^2+^ surge returned to the steady state, CaCl_2_ solution was added (2 mM) to study Ca^2+^ entry. The baselines and areas under the curves of F340/F380 fluorescence were used for comparing the baseline Ca^2+^ levels, SR-Ca^2+^ release, and Ca^2+^ entry between control cells and cells treated with S1.

## Errors in Figures

In the original publication [1], there were errors in Figures 1 and 3–5.

In Figure 1, the graph bars depicting Relative OCR concerning S1 treatment for both 24 h and 72 h were not in the correct position and they have been switched. In Figure 3B, the F340/F380 graph text was wrong. ‘Thapsigargin (100 nM)’ has been replaced with ‘Thapsigargin (2.5 μM)’. In Figure 4A, the images in Control and S1 72 h were identical. The correct images for both Control and S1 72 h were added. Figure 5 presented typographical errors and corrections have been made to correct the text from ‘PCG1’ to ‘PGC-1α’ everywhere it appeared in the figure, as well as “CPT1-B” changed to “CPT1B”. The pAMPKa panel was also misplaced and corrected.

All the corrected figures appear below.

## Error in Figure Legend

In the original publication [1], there was a mistake in the legend for Figure 3. The first phrase of the caption presented errors due to typographical mistakes. The first phrase of the caption has been corrected and appears below under the updated Figure 3.

The authors state that none of the corrections above affect the scientific conclusions. These corrections were approved by the Academic Editor. The original publication has also been updated.

**Figure 1:**
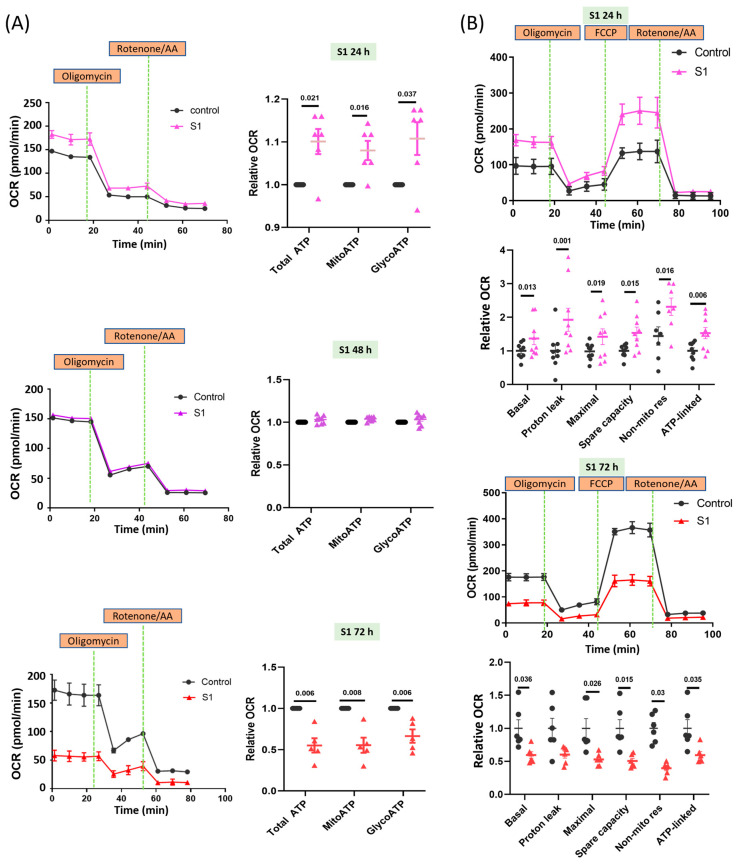
Effects of S1 treatment at different durations on ATP synthesis in AC16 cells. (**A**) Seahorse real-time ATP rate assay showed that ATP production in AC16 cells was increased at 24 h, but reduced at 72 h after S1 (1 nM) treatment (*n* = 6 independent experiments for 24 h, *n* = 8 independent experiments for 48 h, and *n* = 5 independent experiments for 72 h). Total ATP production rate is the sum of the ATP production rate from mitochondrial oxidative phosphorylation and glycolysis. (**B**) Seahorse Mito Stress assay showed that mitochondrial bioenergetics in AC16 cells were increased at 24 h, but decreased at 72 h after S1 (1 nM) treatment (*n* = 9 independent experiments for 24 h and *n* = 6 independent experiment for 72 h). Paired *t*-test was performed for statistical analysis; *p* ≤ 0.05 indicated statistical significance.

**Figure 3:**
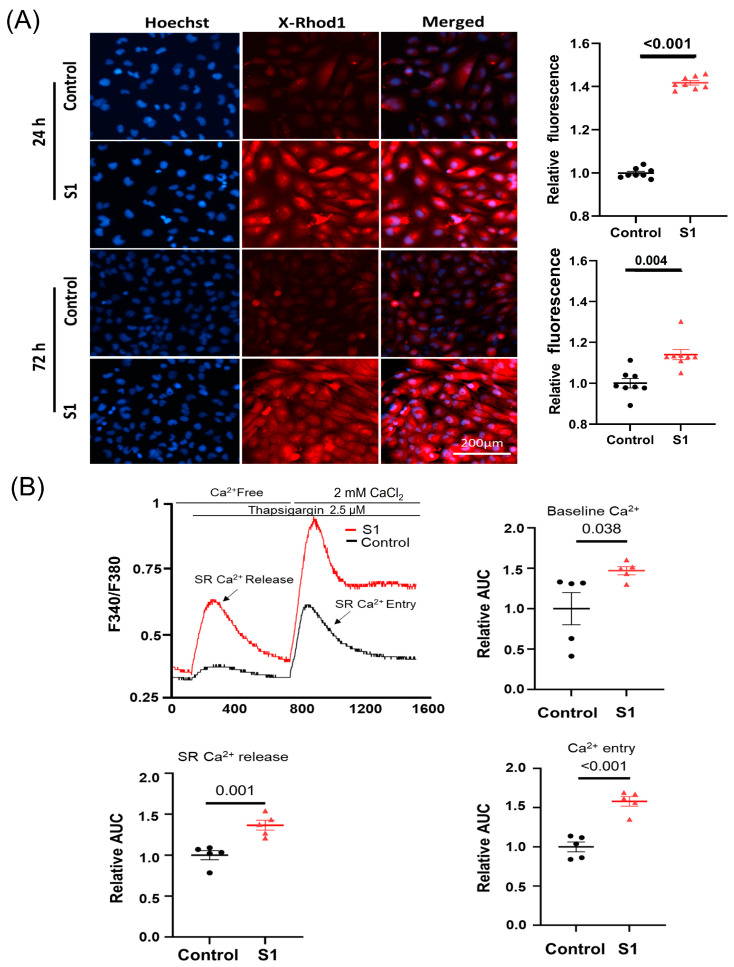
The effects of S1 on AC16 intracellular and mitochondrial calcium (mCa^2+^) levels at 24 h and 72 h post-treatment using Fura-2 and X-Rhod-1 staining, respectively. (**A**) S1 protein increased mCa^2+^ levels in both 24 h- and 72 h-treated cells compared to control groups (*n* = 8 independent experiments). (**B**) S1 protein increased baseline cytosol Ca^2+^ level, SR-Ca^2+^ release, and Ca^2+^ entry (*n* = 5 independent experiments) after treatment for 72 h in AC16. Paired *t*-test was used for comparing control and treated cells.

**Figure 4:**
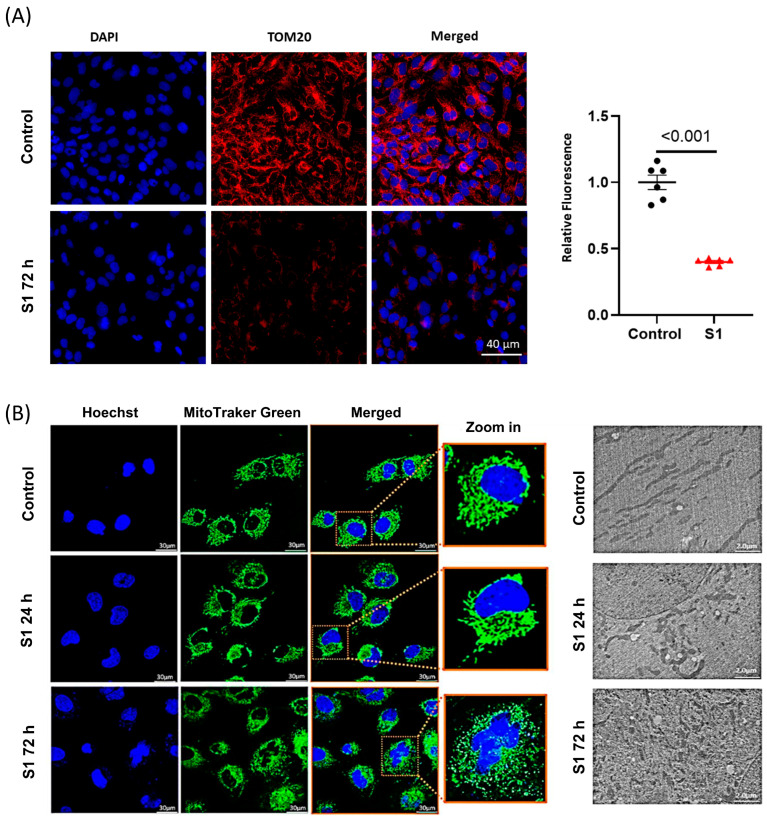
The effects of S1 on mitochondrial dynamics. (**A**) S1 treatment for 72 h decreased TOM20 expression, which is important in transporting essential proteins from cytosol to mitochondria including mitochondrial transcription factors and ETC complex subunits (*n* = 6 independent experiments). (**B**) Mitochondrial morphology evaluated using confocal fluorescence microscopy and transmission electron microscopy. S1 protein disrupted mitochondrial networks and led to mitochondrial fragmentation and mitochondrial fission within 72 h but not within 24 h (*n* = 50 different cells were evaluated for treatment and control).

**Figure 5:**
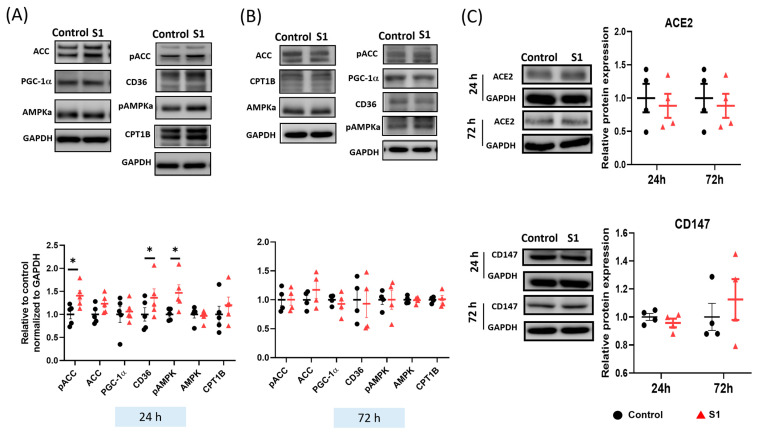
Effects of S1 on the expression of fatty acid transporters and spike protein receptors in AC16 cells after 24 and 72 h treatments. (**A**) S1 (24 h) increased the protein expression of CD36 and phosphorylation of acetyl-coenzyme A carboxylase (ACC) and 5′-adenosine monophosphate–activated protein kinase catalytic subunit alpha-2 (AMPKa), but not the expression of peroxisome proliferator-activated receptor-gamma coactivator-1 and carnitine palmitoyl transferase 1B, total AMPKa, and ACC (*n* = 4–6 independent experiments). (**B**) S1 did not alter the expression of fatty acid transporters at 72 h (*n* = 4 independent experiments). (**C**) S1 did not change protein expression of CD147 and ACE2 of the SARS-CoV-2 receptors at 24 h and 72 h. *n* = 4–6 independent experiments. Paired *t*-test was performed to compare the S1 protein-treated and control cells: * *p* ≤ 0.05.

## References

[1] Huynh T.V., Rethi L., Lee T.-W., Higa S., Kao Y.-H., Chen Y.-J. (2023). Spike Protein Impairs Mitochondrial Function in Human Cardiomyocytes: Mechanisms Underlying Cardiac Injury in COVID-19. Cells.

