# Supplementary material for: Spike Protein Impairs Mitochondrial Function in Human Cardiomyocytes: Mechanisms Underlying Cardiac Injury in COVID-19
Source: Cells. 2023 Mar 11;12(6):877. doi: 10.3390/cells12060877 (PMC10046940; doi:10.3390/cells12060877)
Supplement: Supplementary file 1 [file cells-12-00877-s001.zip › cells-2249211-supplementary.pdf]

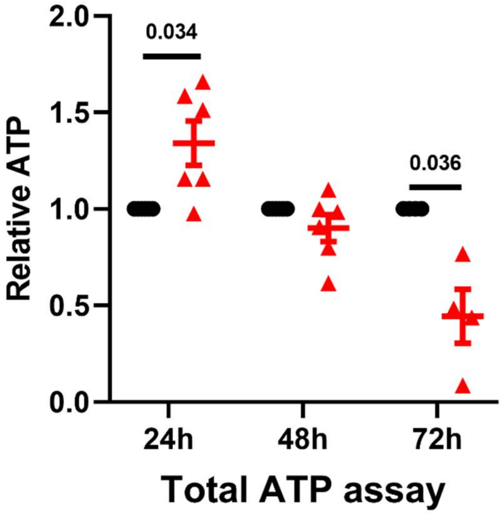

Supplementary Figure S1. S1 on total cellular ATP production in AC16 cells. Total cellular ATP in AC16 cells was increased at 24 h, but decreased at 72 h after S1 (1 nM) treatment (n = 6 independent experiments for 24 h and 48 h; n = 4 independent experiment for 72 h). Paired *t* test was performed for statistical analysis; $p \leq .05$ indicated statistical significance.

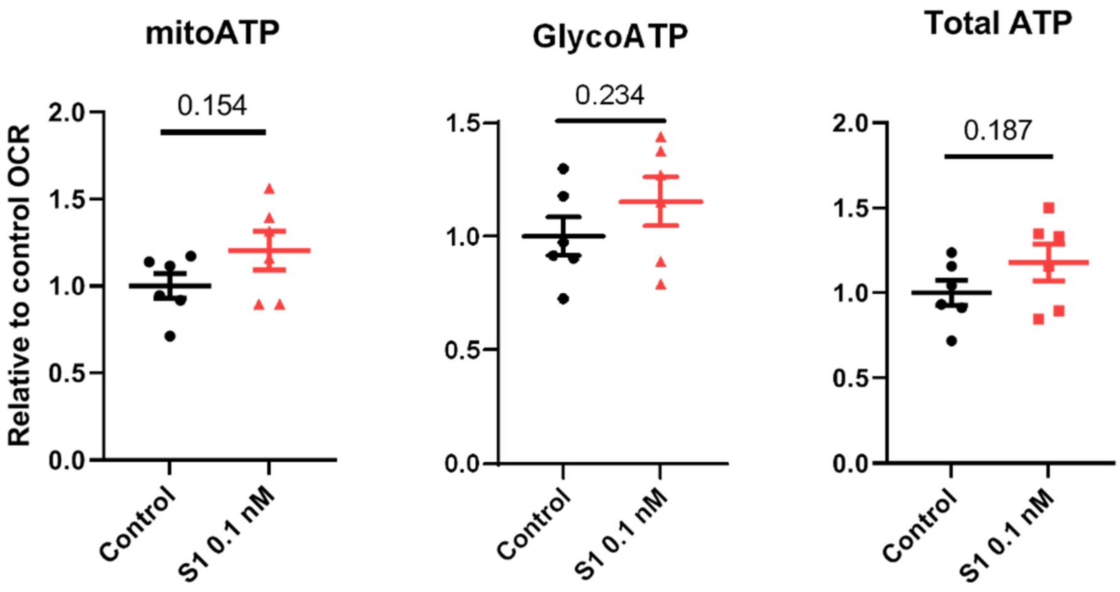

Supplementary Figure S2. S1 at lower concentration (0.1 nM) on ATP production rate in AC16 cells. ATP production rate from glycolysis and mitochondrial oxidative phosphorylation was not changed after treatment with S1 for 24 h (n = 6 independent experiments).
